# P-1246. Evaluation of the Effectiveness of Administering Posaconazole and Isavuconazole via Enteral Feeding Tubes

**DOI:** 10.1093/ofid/ofaf695.1438

**Published:** 2026-01-11

**Authors:** Mallory Yan, Blake Williams, Emir Kobic, Michelle Potter

**Affiliations:** Banner University Medical Center - Phoenix, Phoenix, Arizona; University of Arizona R. Ken Coit College of Pharmacy, Phoenix, Arizona; Banner University Medical Center Phoenix, Phoenix, Arizona; Banner University Medical Center Phoenix, Phoenix, Arizona

## Abstract

**Background:**

Invasive fungal infections are a significant source of morbidity and mortality in immunocompromised and critically ill patients. Posaconazole and isavuconazole are commonly used for prophylaxis and treatment of invasive fungal infections in these populations. Oral therapy has many advantages over IV, including convenience and decreased IV-associated complications. Limited studies exist assessing the appropriateness of administering these medications via enteral feeding tube (EFT), but recent case series show feasibility in achieving therapeutic levels.

**Table 1.**
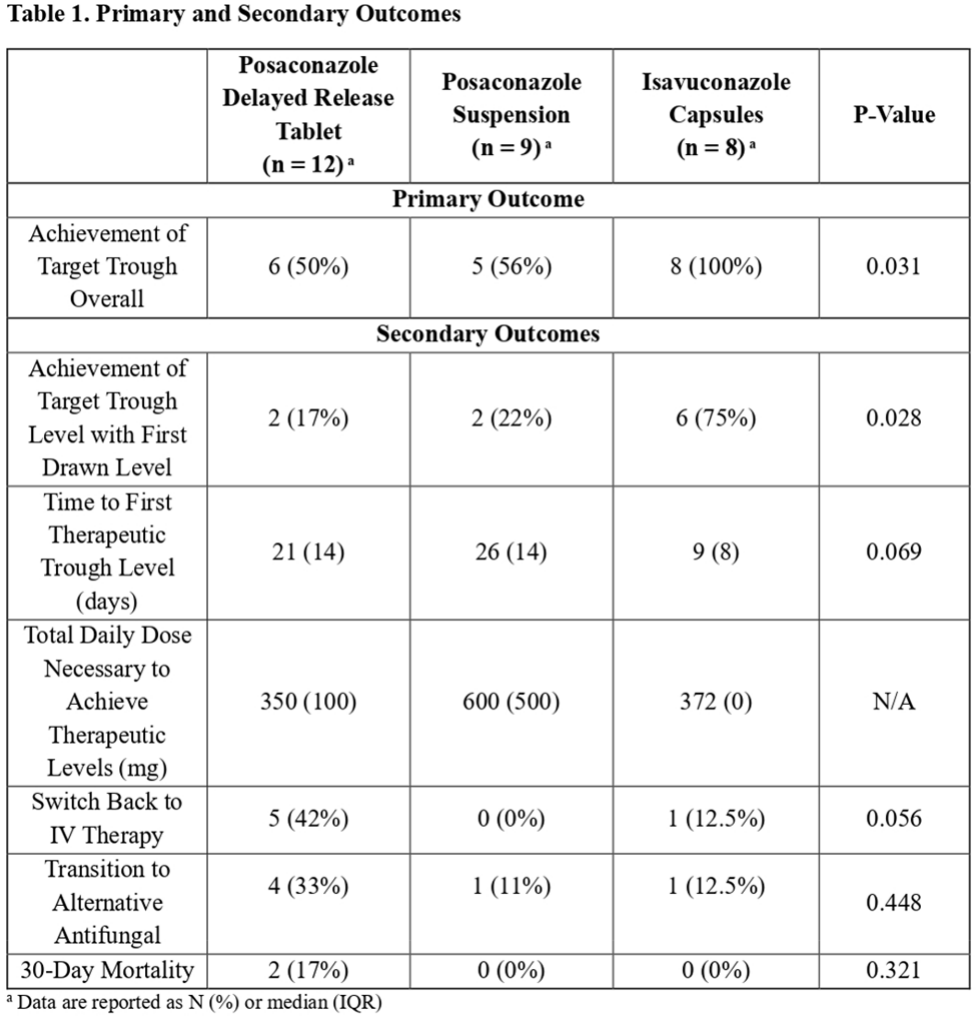
Primary and Secondary Outcomes

**Methods:**

A single center retrospective chart review was performed for adult patients who received posaconazole or isavuconazole via EFT between January 1^st^ 2020 and August 31^st^ 2024. Key exclusion criteria included patients who received the antifungals via other routes of administration, no trough levels, or non-true trough levels (drawn too early or drawn before five days of therapy). For statistical analysis, continuous variables were reported as medians with interquartile ranges and compared using Mann-Whitney U tests. Categorical variables were reported as counts and percents and were compared utilizing Fisher’s exact tests. All statistical comparisons used a two-tailed alpha of 0.05.

**Results:**

Eighty-six patients were reviewed for inclusion, and 29 patients were included in the final data analysis. Forty-nine percent of patients were receiving these medications for prophylaxis, and the remainder for treatment. Overall achievement of target trough levels was successful in 50% of patients who received posaconazole delayed release tablets (DRT), 56% of patients that received posaconazole suspension, and 100% of patients that received isavuconazole (p = 0.031; Table 1). Posaconazole patients also had lower rates of target trough attainment with first level drawn and took longer to achieve the first therapeutic trough level. No differences in 30-day mortality were found between treatment groups.

**Conclusion:**

This study suggests that isavuconazole capsules, but not posaconazole, can be administered via EFT with adequate level attainment. Crushed posaconazole in DRT or suspension form was significantly less likely to reach therapeutic trough levels compared to isavuconazole when administered through EFT.

**Disclosures:**

Emir Kobic, BCIDP, Shionogi: Honoraria

